# Psychometric evaluation and measurement invariance of the Sexual and Relationship Distress Scale in cancer and nonclinical general reproductive-age populations

**DOI:** 10.1093/sexmed/qfaf041

**Published:** 2025-06-02

**Authors:** Yanfei Jin, Yang Li, Lina Xiong, Chulei Tang, Hongwen Ma

**Affiliations:** Department of Clinical Nursing, School of Nursing, Nanjing Medical University, Nanjing 211166, Jiangsu, China; Department of Nursing, Tianjin Union Medical Center, The First Affiliated Hospital of Nankai University, Hongqiao District, Tianjin 300070, China; Department of Orthopedics, The First Affiliated Hospital of Nanchang University, Nanchang 330209, Jiangxi, China; Department of Clinical Nursing, School of Nursing, Nanjing Medical University, Nanjing 211166, Jiangsu, China; Department of Nursing, Tianjin Union Medical Center, The First Affiliated Hospital of Nankai University, Hongqiao District, Tianjin 300070, China

**Keywords:** cancer, nonclinical general population, psychometric evaluation, reproductive age, sexual and relationship distress

## Abstract

**Background:**

The Sexual and Relationship Distress Scale (SaRDS) is a validated instrument developed in English to assess intra-personal and inter-personal distress experienced by individuals and their partners in the context of sexual dysfunction. However, it has not yet been translated into Chinese nor psychometrically evaluated within Chinese clinical cancer and nonclinical populations.

**Objective:**

This study aimed to translate the SaRDS into Chinese and assess its psychometric properties and measurement invariance across different populations (colorectal cancer [CRC] patients vs. nonclinical general reproductive-age adults) and across gender groups (male vs. female).

**Methods:**

Three phases were undertaken: (a) transcultural adaptation, (b) pre-testing, and (c) psychometric evaluation. Transcultural adaptation included translations and expert panels, the pre-testing was conducted in 20 participants. The psychometric evaluation was tested among 486 CRC patients and 536 nonclinical general reproductive-age populations.

**Outcomes:**

The Chinese version of the SaRDS was consistent with the original version, including 30 items and 14 factors.

**Results:**

Confirmatory factor analysis supported the 14-factor structure of the original SaRDS construct. The composite reliability and the average variance extracted indicated the SaRDS had good convergent validity. Measurement invariance analyses indicated that the factor structure, factor loadings, and item intercepts of the SaRDS were invariant across CRC and nonclinical general populations, as well as across gender groups. The correlation of SaRDS with the Arizona Sexual Experience Scale and the Quality of Relationship Index showed good criterion-related validity. Moreover, the SaRDS and subscales had high internal consistency.

**Clinical implications:**

The Chinese version of the SaRDS is a psychometrically robust tool suitable for evaluating individual and relationship distress related to sexual dysfunction among clinical cancer and nonclinical general populations. The 14 domains provided by the SaRDS enable clinicians to identify specific areas of distress, facilitating accurate assessment and tailored interventions for individuals and couples experiencing sexual difficulties.

**Strengths and limitations:**

This study provides the first evidence of measurement invariance of the SaRDS across cancer patients, nonclinical general populations, and gender groups. However, due to its cross-sectional design, future longitudinal studies are needed to further examine the temporal stability and measurement invariance over time.

**Conclusion:**

Our findings suggest that the Chinese version of the SaRDS is a reliable, valid, and psychometrically sound instrument for assessing sexual and relationship distress in clinical cancer and nonclinical reproductive-age populations. Its demonstrated measurement invariance across populations and genders supports its broad applicability in clinical practice and research.

## Introduction

Personal distress is a necessary condition to diagnose most mental health problems in clinical practice. To make a diagnosis, it is required that there should be not only the presentation of a series of corresponding symptoms and signs but also the individual’s experience of distress to accompany these symptoms and signs.^1^ Sexual dysfunctions (eg, female genito-pelvic pain/penetration disorder, male erectile disorder/premature ejaculation, etc.) constitute a spectrum of sexual function problems whose core characteristic is the experience of sexual distress alongside sexual functioning. Therefore, the Diagnostic and Statistical Manual of Mental Disorders, Fifth Edition (DSM-5), makes the presence of “clinically significant distress” central to the definition and diagnosis of sexual dysfunction.^2^

It is important to note that clinically significant distress associated with sexual dysfunction and sexual distress more broadly are related but distinct constructs. Clinically significant distress, as defined by DSM-5, specifically refers to the individual's distress directly linked to the presence and perceived severity of sexual dysfunction symptoms, and it is a requisite criterion for diagnosing sexual dysfunction. In contrast, sexual distress can include a wide range of negative emotional experiences related to sexuality, intimacy, and relational contexts, which may or may not directly result from specific sexual dysfunction symptoms.

Sexual distress is one of the most important factors in the etiology, maintenance, and treatment of sexual dysfunction. Neglecting sexual distress may lead to inaccurate measurement of sexual dysfunction, and reduction in functional symptoms alone is not sufficient to reduce individual distress about sexual dysfunction. However, not all studies have considered sexual distress caused by sexual dysfunction, which is not only reflected in the fact that many researchers have not strictly distinguished between sexually impaired function (distress is not present) and sexual dysfunction (both impaired function and distress are present) according to the requirements of the DSM,^3^ but also in the lack of tools to measure sexual distress. Measures that assess sexual distress in a comprehensive, valid, and precise manner are necessary to evaluate and compare sexual outcomes and to develop interventions for those who experience or are at risk for sexual dysfunction. Indeed, the need for high-quality patient-reported outcome measures (PROMs) of sexual distress has been repeatedly emphasized by relevant international sexual function research panels and society.^4^^,^^5^

Sexual functioning occurs most commonly in a dyadic context. According to the interdependence theory, individuals influence each other’s experiences (emotions, motivations, behaviors, and outcomes) through interactions.^6^ This theory emphasizes that relationship factors and processes play a crucial role in the quality of sexual response. Being in a partnership appears to be an important factor in explaining the presence of distress from sexual dysfunction.^7^ Still, the relational context has historically been ignored by most research on the causes and treatments of sexual dysfunction. Research has confirmed that relationship is one of the strongest predictors of sexual distress, meaning that individuals with high relationship satisfaction experience lower levels of sexual distress.^8^^,^^9^ Moreover, in intervention studies, there is evidence to suggest that taking a couples approach to the treatment of sexual dysfunction is beneficial and relationship satisfaction is a significant moderator of sexual distress.^10^^,^^11^ Given that impaired sexual function has historically been difficult to treat, reducing sexual distress alone may be an achievable treatment goal that is effective in promoting sexual outcomes.^12^ Thus, it is important to develop a measure of sexual distress that both members of the couple can use in research and clinical settings.

Previously, due to a lack of attention to sexual distress at both intra-personal and inter-personal levels, there were limited measurement tools for sexual and relationship distress, with the only available and validated tool being the Sexual Desire and Relationship Distress Scale (SDRDS).^13^ However, the SDRDS is only specifically applicable to women with low sexual desire. To expand sexual distress from the intra-personal to the relationship level, Frost and Donovan constructed the Sexual and Relationship Distress Scale (SaRDS) to accurately capture both the intra-personal and inter-personal distress experienced by individuals in a relationship partner with sexual dysfunction.^14^ The SaRDS includes multiple dimensions to quantify different types of distress; while also can use a total score to provide more information on the outcome efficacy. It was developed rigorously and has shown acceptable internal consistency, convergent validity, discriminant validity, and construct validity.

Given that clinically significant distress associated with sexual dysfunction is central to the diagnosis and clinical management of sexual dysfunction, and recognizing the broader role that sexual and relationship distress play in affecting sexual outcomes, we believe that effective measurement of these forms of distress is of great significance. To our knowledge, there is currently no comprehensive, valid, and reliable instrument in China capable of capturing sexual and relationship distress simultaneously among adult men and women. To promote clinical research and practice addressing sexual dysfunction and related distress in China, the current study aimed to translate the SaRDS into Chinese and evaluate its psychometric properties among Chinese adults.

As the third largest cancer in the world, colorectal cancer (CRC) has a unique treatment combination of abdominoperineal resection, pelvic radiotherapy, and chemotherapy, which significantly increases the incidence and severity of sexual dysfunction in CRC patients compared to other cancer types.^15–17^ Notably, the incidence of CRC among reproductive-aged adults (15–49 years old) is increasing rapidly.^18^^,^^19^ Compared with elderly CRC patients and normal peers, patients of reproductive age face more severe challenges, with more than 50% of CRC patients of reproductive age reporting that sexual dysfunction had a serious negative impact on their own and their partners’ intimate relationships and quality of life.^15^^,^^20^

## Aims

Therefore, it is crucial to develop and validate instruments that can reliably assess sexual and relationship distress across diverse populations and genders. To verify the broad applicability and robustness of the SaRDS, the overall objective of the present study was to evaluate the psychometric properties and measurement invariance of the Chinese version of the SaRDS among CRC patients and nonclinical reproductive-aged adults, and across gender groups. Specifically, this study aimed to address two primary research questions: (1) Does the Chinese version of the SaRDS demonstrate adequate psychometric properties (validity and reliability) among CRC patients and nonclinical reproductive-aged adults? (2) Does the Chinese version of the SaRDS exhibit measurement invariance across population groups (CRC patients vs. nonclinical reproductive-aged adults) and across gender groups (male vs. female)?

## Methods

### Design

This study was designed as a cross-sectional survey and three distinct phases were undertaken: (1) transcultural adaptation, (2) pre-testing, and (3) psychometric evaluation. The reporting of this study adhered to the Strengthening the Reporting of Observational Studies in Epidemiology (STROBE) guidelines, and a completed STROBE checklist has been submitted with this manuscript.

### Phase 1: Transcultural adaptation

The standardization procedure of the transcultural adaptation was guided by the World Health Organization’s Process of Translation and Adaptation of Instrument.

#### Forward translation

Permission to translate and validate the SaRDS was obtained from the copyright holder. Forward translation of the SaRDS was separately delegated to two bilingual translators to translate the original SaRDS from English to Chinese. Both of them had a medical background, one translator was aware of the purpose of the SaRDS translation, whereas the other was not. After the two translators finished the initial translation, item discrepancies between the two versions were identified and resolved, and the two versions were merged into a single version.

#### Backward translation

Subsequently, another two bilingual translators (who were unaware of the study’s purposes and measures) did a back translation from the Chinese version of the SaRDS into English. The back-translated version was remarkably similar to the original SaRDS. Then, the pre-final version was developed.

#### Expert panels

Five psychologists served as the expert group that assessed the SaRDS in its pre-final form. Three questions were addressed, namely: (1) Is the Chinese word that has been translated exactly the same as the English word? (2) Is there a better Chinese word to utilize as an alternative if the translated word is not exactly the same? (3) How likely is it that those who speak Chinese and fill out the questionnaire will be able to understand the substitute word that is selected? The purpose of this step is to review discrepancies in the meanings of the scale items and evaluate the cultural and linguistic equivalence of each item until a consensus is achieved.

The face validity and content validity of the SaRDS were then assessed by a second expert panel, which was made up of three sexual and reproductive doctors, three associate professors, and four nursing specialists. Wording, grammar, and item placement were used to evaluate face validity, while a Content Validity Index (CVI) was used to evaluate content validity. The CVI was completed by the item relevance ratings (1 = *not relevant*; 4 = *highly relevant*) of the expert panel,^21^ which included content validity scores for each item-level (I-CVI) and a scale-level CVI/average (S-CVI/Ave). The I-CVI is the proportion of experts who rate an item as relevant, whereas the S-CVI/Ave is the proportion of items rated as relevant by all raters. For a scale to be judged as having excellent content validity, it should contain only items with an I-CVI of ≥.78 and a S-CVI/Ave of ≥.90.^22^

### Phase 2: Pre-testing

The pre-final version was pre-tested in 20 participants (10 CRC and 10 nonclinical general reproductive-age adults each). The inclusion criteria for patients were as follows: (1) Diagnosed with CRC, with a duration of at least 3 months after surgery; (2) aged 18-49 and married, (3) were able to complete the questionnaire independently, and (4) were willing to participate. The inclusion criteria for nonclinical populations were as follows: (1) Aged 18-49 and married, (2) were able to complete the questionnaire independently, and (3) were willing to participate. The exclusion criteria for both CRC patients and nonclinical populations were cognitive or psychiatric impairments and suffering from severe heart, liver, kidney, and other serious complications. Participants were asked to comment on their understanding of the wording and report on the clarity of the items.

### Phase 3: Psychometric evaluation

#### Setting and participants

The CRC and nonclinical general reproductive-age populations were recruited from tertiary hospitals and communities in Tianjin and Jiangxi, China, from March 2024 to June 2024, respectively. The inclusion and exclusion criteria for participants are the same as those for pre-testing.

### Measures

#### Demographics and clinical information

Demographic and clinical information were obtained from the participants using a questionnaire designed by the researcher that yielded information about age, gender, education, marital status, number of children, and clinical relevance.

### The SaRDS

The SaRDS is a 30-item, 14-factor PROM of individual and relationship distress within the context of sexual dysfunction.^14^ All items are rated on a 7-point Likert scale (“not at all true” to “completely true”). The total scores range from 0 to 180 and reflect the standard of individual and relationship distress within the context of sexual dysfunction, with high values indicating more distress. In the original version of the study, the SaRDS showed good psychometric properties, and the subsequent studies also reported the strong internal consistency of the SaRDS (Cronbach *α* = .95 for the total score with individual subscales ranging from .70 to .96).

### The Arizona Sexual Experience Scale

The Arizona Sexual Experience Scale (ASEX) is a 5-item scale that quantifies sex drive, arousal, vaginal lubrication/penile erection, ability to reach orgasm, and satisfaction from orgasm.^23^ All items are rated on a 6-point Likert scale (“extremely strong” to “no sex drive”). Total scores range from 5 to 30, and higher scores denote more sexual dysfunction. The internal consistency of the Chinese version of ASEX was *α* = .855.

### The Quality of Relationship Index

The Quality of Relationship Index (QRI) is a 6-item measure to assess the quality of relationships in married and cohabiting couples.^24^ All items are rated on a 7-point Likert scale (“extremely dissatisfied” to “extremely satisfied”). The total scores range from 7 to 42, and higher scores denote greater relationship satisfaction. The internal consistency of the Chinese version of QRI was *α* = .782.

### Data collection

The research team consists of an associate professor as the leader, two master students, and two registered nurses as research assistants, all of whom have been systematically trained. Recruitment was conducted by research members who approached the potential participants when they entered the hospitals (outpatient or inpatient departments) and communities. In both locations, research members presented the content and purpose of the research to potential participants, assessed them to determine whether they met the inclusion and exclusion criteria, and assured that participation was voluntary. After obtaining written consent, the researcher distributed the questionnaires to the participants. Once they completed the questionnaire, the researcher immediately checked their questionnaire to ensure the integrity of the data. If there were missing values, the questionnaires were returned to the participants so that they could fill in the missing items. Generally, the questionnaires were completed within 10–15 min.

### Ethical considerations

This study was approved by the Institutional Review Board of Nanjing Medical University (Ethics No. 2024-749). All participants provided written informed consent prior to participation, and they were explicitly informed of their right to withdraw from the study at any point without any prejudice or consequences.

### Statistical analyses

The data analysis was performed using IBM SPSS Statistics Version 26.0 (IBM Corp., Armonk, NY, USA) and Analysis of Moment Structure Version 23.0 (IBM Corp., Armonk, NY, USA).

First, descriptive statistics (frequency, percentages, means, and standard deviations) were used to describe the demographic characteristics of participants. Prior to conducting analyses, data were carefully screened for missing values. In our dataset, the proportion of missing data was very small (<5% per variable), and the missing pattern appeared completely at random (MCAR). Therefore, missing data were handled using expectation–maximization imputation, which is considered a robust and recommended method for addressing missing values under the assumption of MCAR.

Confirmatory factor analysis (CFA) was used as a critical step in refining the instrument and identifying the factorial structure of individual and relationship distress within the context of sexual dysfunction in the SaRDS. The CFA was conducted using the Maximum Likelihood estimation method, which is the most widely used method for parameter estimation in CFA due to its robustness and accuracy when data meet assumptions of approximate multivariate normality. Four fit indices were employed to examine the adequacy of model fit: a Chi-square to degrees of freedom ratio (*χ*^2^/*df* < 3), the comparative fit index (CFI ≥ 0.90), the Tucker–Lewis index (TLI ≥ 0.90), and the root mean square error of approximation (RMSEA ≤ 0.05).^25^ In CFA, the convergent validity of the SaRDS was verified through the factor loading of each item (≥0.50),^26^ the composite reliability (CR) value (≥0.70), and the average variance extracted (AVE) of the 14 factors (≥0.50).^27^

Then, to establish whether the 14-factor model is stable and can be replicated across different groups (CRC and nonclinical general populations), measurement invariance was further evaluated using multiple-group CFA. To examine whether the 14-factor model structure of the SaRDS is stable and replicable across different populations (CRC patients vs. nonclinical general populations) and across gender groups (male vs. female), we conducted multi-group CFA to systematically evaluate measurement invariance. The significance test of the change in Chi-square for two nested models was evaluated as a criterion for measurement invariance. If the Chi-square is not significant, it is determined that factor loadings are equivalent and thus evidence of weak metric invariance and scalar invariance.^28^

Furthermore, criterion-related validity was measured by the Pearson correlation between SaRDS and ASEX and the Pearson correlation between SaRDS and QRI, respectively. Last, to evaluate the internal consistency of the SaRDS, Cronbach’s *α* was measured. Internal consistency was considered adequate when *α* ≥ .70.^29^

## Result

### Phase 1: Transcultural adaptation

#### Forward translation

In the step of forward translation, the English version was translated into Chinese. The two translators only had discrepancies in the translation of the two items under “Dimension 6: Security” and reached an agreement after discussion.

#### Backward translation

In the step of backward translation, the Chinese version was translated back into English. The back-translated version was remarkably similar to the original SaRDS.

#### Expert panels

The first expert panel unanimously approved the translation and original review of SaRDS. However, the experts thought that items 13 and 14 were ambiguous, so the wording of items 13 and 14 was modified after discussion. In addition, the experts thought that item 15 was difficult to understand, so item 15 was also appropriately modified. In the second panel, the 10 experts gave their comments on the scale and rated each item’s understandability, clarity, sensitivity, and relevance. The I-CVIs ranged from 0.87 to 1.00, and the calculated S-CVI was 0.95, indicating that the SaRDS content was valid.

### Phase 2: Pre-testing

In this step, some patients pointed out that the description of item 20 was unclear. After discussion, the wording of item 20 was modified. After the revision, the 20 participants reported that all items were clear, understandable, and acceptable. Thus, the final Chinese version of the SaRDS was generated (The 30 SaRDS items (English and Chinese versions) are available in Supplementary Table S1).

### Phase 3: Psychometric evaluation

#### Sample characteristics

Of the 1211 potential participants that were approached, 104 did not meet the inclusion criteria, 64 declined to participate for various reasons, and 1043 were eligible for the study and consented to participate. Finally, 1022 remained (486 CRC and 536 nonclinical general population) after the invalid questionnaires were removed, with no missing values. Characteristics of the participants are presented in Table 1, and descriptive statistics of SaRDS in the CRC and nonclinical general reproductive-age populations are presented in Table 2.

**Table 1:** Demographic and clinical characteristics of the samples (*N* = 1022).

**Variable**	**CRC population (*N* = 486)**	**General population (*N* = 536)**
**Age (mean ± SD)**	37.124 ± 3.1110	34.978 ± 4.970
Gender		
Male	287 (59.05)	214 (39.93)
Female	199 (40.95)	322 (60.07)
**Education**		
Primary or blew	45 (9.26)	109 (20.34)
Junior high school	155 (31.89)	176 (32.84)
Senior high school	196 (40.33)	181 (33.77)
College and above	90 (18.52)	70 (13.06)
**Partner’s education**		
Primary or blew	83 (17.08)	73 (13.62)
Junior high school	176 (36.21)	167 (31.16)
Senior high school	148 (30.45)	210 (39.18)
College and above	79 (16.26)	86 (16.04)
**Monthly income (yuan)**		
<3000	104 (21.40)	135 (25.19)
3001-5000	296 (60.91)	244 (45.52)
>5000	86 (17.70)	157 (29.29)
**Place of residence**		
Rural	306 (62.96)	
City	180 (37.04)	
**Number of children**		
None	75 (15.43)	
≥1	411 (84.57)	
**Family history of CRC**		
Yes	58 (11.93)	
No	428 (88.07)	
**Chemotherapy or radiation**		
Yes	327 (67.28)	
No	159 (32.72)	
**Complications of stoma**		
Yes	321 (66.05)	
No	165 (33.95)	
**Comorbidity**		
Yes	132 (27.16)	
No	354 (72.84)	
**Existence of stoma**		
Yes	198 (40.74)	
No	288 (59.26)	

Abbreviations: CRC = colorectal cancer; SaRDS = Sexual and Relationship Distress Scale.

**Table 2:** Descriptive statistics of SaRDS in CRC population and nonclinical general population.

**Subscales**	**Group**	**Mean**	***SD***	***t***	***P***
1. Anxiety	1	11.64	4.44	12.84	<.001
	2	8.66	2.88		
2. Conflict	1	12.00	4.03	15.24	<.001
	2	8.66	2.92		
3. Initiation	1	8.21	3.19	13.32	<.001
	2	6.01	2.03		
4. Guilt	1	8.21	2.71	14.70	<.001
	2	6.13	1.75		
5. Infidelity	1	8.74	2.54	23.31	<.001
	2	5.45	1.96		
6. Security	1	8.12	3.23	11.74	<.001
	2	6.17	1.97		
7. Predictability	1	8.76	2.68	18.18	<.001
	2	6.20	1.78		
8. Communication	1	8.92	2.46	23.46	<.001
	2	5.72	1.89		
9. Body image	1	8.62	3.14	16.01	<.001
	2	6.00	2.02		
10. Physical affection	1	7.78	3.34	11.31	<.001
	2	5.84	2.04		
11. Hopelessness	1	8.27	3.18	11.35	<.001
	2	6.46	1.78		
12. Self-esteem	1	8.21	3.29	11.95	<.001
	2	6.16	2.11		
13. Normalness	1	8.72	2.97	16.62	<.001
	2	6.10	2.01		
14. Relationship quality	1	8.95	2.98	17.44	<.001
	2	6.12	2.18		
Total score	1	125.15	28.22	22.91	<.001
	2	89.69	20.16		

*Note*. Group 1 = CRC population; Group 2 = nonclinical general population. Abbreviations: CRC = colorectal cancer; SaRDS = Sexual and Relationship Distress Scale.

### Confirmatory factor analysis

**Figure 1:**
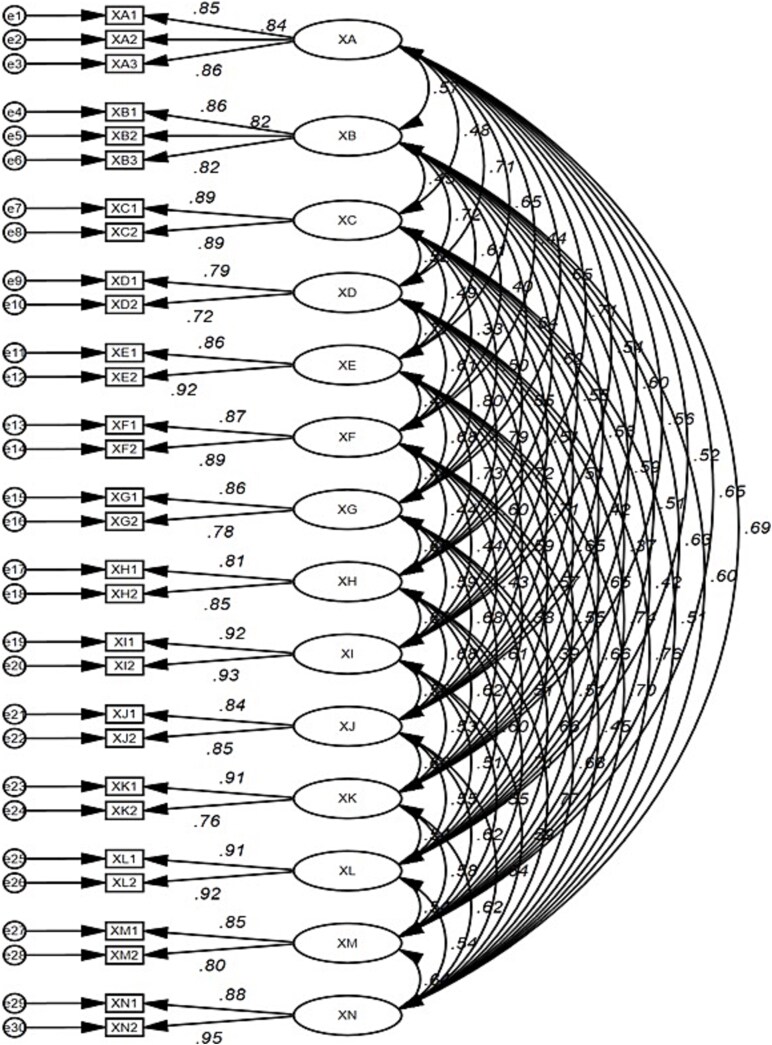
Factor structure of the refined model of the SaRDS. XA = anxiety; XB = conflict; XC = initiation; XD = guilt; XE = infidelity; XF = security; XG = predictability; XH = communication; XI = body image; XJ = physical affection; XK = hopelessness; XL = self-esteem; XM = normalness; XN = relationship quality; SaRDS = Sexual and Relationship Distress Scale.

The CFA model with 14 latent subscales demonstrated an adequate fit across multiple fit indices. Final fit statistics were all optimal as follows: Chi-square (*χ*^2^/*df* = 2.496, *P* < .001), TLI = 0.971, CFI = 0.979, RMSEA = 0.037. Figure 1 shows the results of the factor structure and model fit of the SaRDS using CFA.

The convergent validity of the SaRDS was examined by CFA as well as AVE and CR. The results showed that the factor loadings of all items ranged from 0.725 to 0.950, the AVE values of 14 factors ranged from 0.574 to 0.857, and the CR values ranged from 0.729 to 0.923. As described earlier, all of the criteria displayed good acceptability, supporting that the SaRDS has satisfactory convergent validity (Table 3).

**Table 3:** Factor structures by confirmatory factor analysis (*N* = 1022).

**Items**	**Factor**	***SE***	***P***	**CR**	**AVE**
1. I worry about sex even when I am not with my partner	Anxiety	0.848	<.001	0.886	0.722
2. I feel anxious when I think about our sexual relationship		0.838	<.001		
3. I am stressed about sex		0.863			
4. My partner and I get angry with each other	Conflict	0.858	<.001	0.871	0.693
5. My partner and I regularly argue		0.820	<.001		
6. My partner and I get annoyed with each other over little things		0.819			
7. I do not initiate sex with my partner anymore	Initiation	0.887	<.001	0.881	0.787
8. I rarely bother to approach my partner for sex		0.887			
9. I feel guilty because I cannot sexually satisfy my partner	Guilt	0.789	<.001	0.729	0.574
10. I feel guilty for letting my partner down		0.725			
11. I am worried that my partner has been unfaithful	Infidelity	0.865	<.001	0.887	0.797
12. I am worried that my partner will be unfaithful		0.920			
13. I feel undesirable to my partner	Security	0.870	<.001	0.872	0.773
14. I feel unattractive to my partner		0.889			
15. Our sex is routine or predictable	Predictability	0.859	<.001	0.803	0.671
16. There is not much variety when we have sex		0.778			
17. My partner and I do not talk about sex	Communication	0.809	<.001	0.815	0.689
18. I avoid talking about sex with my partner		0.850			
19. I am worried that our relationship might end	Body image	0.922	<.001	0.923	0.857
20. I am questioning the strength of our relationship		0.930			
21. We don’t hug and kiss as much as we used to	Physical affection	0.843	<.001	0.835	0.716
22. We are not as physically affectionate as we used to be		0.849			
23. I wish more effort was made to fix our sexual problems	Hopelessness	0.914	<.001	0.829	0.709
24. I feel frustrated that I can’t fix our sexual problems		0.764			
25. I have lower confidence because of our sexual problems	Self-esteem	0.909	<.001	0.911	0.837
26. I have lower self-esteem because of our sexual problems		0.920			
27. My relationship has become more like a friendship	Normalness	0.846	<.001	0.810	0.681
28. My partner and I feel more like flat mates or colleagues		0.804			
29. I worry there is something wrong with me sexually	Relationship quality	0.876	<.001	0.910	0.835
30. I do not feel normal when I compare myself sexually to others		0.950	<.001		

Abbrevations: *SE* = standardized estimate; CR = composite reliability; AVE = average variance extracted.

Furthermore, we conducted multi-group confirmatory factor analyses to examine the measurement invariance of the SaRDS across different population groups and gender groups. As shown in Table 4, all tested invariance models exhibited moderate-to-good fit indices. Specifically, the SaRDS demonstrated satisfactory configural, metric, and scalar invariance both across different population groups and across gender groups. These findings indicate that the factor structure, factor loadings, and item intercepts of the SaRDS are equivalent across populations and genders, supporting the robustness and generalizability of the scale.

**Table 4:** Model fit of various invariance models (*N* = 1022).

**Model**	${\mathrm{\chi}}^{\mathbf{2}}$	$\boldsymbol{df}$	**CFI**	**TLI**	**RMSEA**	${\boldsymbol{\Delta} \mathrm{\chi}}^{\mathbf{2}}$	**Δ** $\boldsymbol{df}$	***P***
**Population**								
1. Configural invariance	970.342	628	0.980	0.972	0.033			
2. Metric invariance	980.998	644	0.979	0.973	0.034	10.657	16	.830
3. Scalar invariance	1119.763	660	0.973	0.964	0.037	149.421	32	.000
**Gender**								
1. Configural invariance	1114.435	628	0.977	0.968	0.039			
2. Metric invariance	1137.120	644	0.978	0.969	0.038	22.685	16	.122
3. Scalar invariance	1119.763	660	0.981	0.970	0.036	12.358	16	.719

Abbreviations: RMSEA = root mean square error of approximation; CFI = comparative fit index; TLI = Tucker–Lewis index.

### Criterion-related validity

We used ASEX and QRI as gold standards to test the criterion-related validity of SaRDS. The results showed that SaRDS had a significant weak positive correlation with ASEX (*r* = 0.230, *P* < .001); and a significant moderate negative correlation with QRI (*r* = −0.625, *P* < .001), indicating adequate criterion-related validity of the SaRDS (Table 5).

**Table 5:** Correlation of the SaRDS with the ASEX and QRI, and Cronbach’s *α* (*N* = 1022).

**SaRDS and subscales**	**ASEX** ***r* (***P***)**	**QRI** ***r* (***P***)**	**Cronbach’s** $\boldsymbol{\alpha}$
SaRDS	.230^***^	−.625^***^	0.865
1. Anxiety			0.870
2. Conflict			0.881
3. Initiation			0.702
4. Guilt			0.892
5. Infidelity			0.885
6. Security			0.798
7. Predictability			0.711
8. Communication			0.813
9. Body image			0.925
10. Physical affection			0.833
11. Hopelessness			0.806
12. Self-esteem			0.912
13. Normalness			0.810
14. Relationship quality			0.958

Abbreviations: SaRDS = Sexual and Relationship Distress Scale; ASEX = Arizona Sexual Experience Scale; QRI = Quality of Relationship Index. ^*^P < .05; ^**^P < .01; ^***^P < .001.

### Internal consistency

To determine internal consistency, Cronbach’s *α* values were calculated individually for the subscales of the SaRDS. Cronbach’s *α* values for the 14 subscales ranged from .702 to .958, indicating that the SaRDS has good internal consistency (Table 5).

## Discussion

Given the growing body of research viewing distress from sexual dysfunction as an issue that often exists within the context of a relationship, an adequate tool for measuring sexual and relationship distress is of great significance. This study translated the SaRDS into Chinese and evaluated the psychometric properties of the Chinese version of the SaRDS in both CRC and nonclinical reproductive-age samples in China. The translation of SaRDS in this study involves two languages from different linguistic groups. The source language was English, which belongs to Indo-European, while the target language was Chinese, a Sino-Tibetan language. Due to the huge differences in cultural background, these two languages differ semantically, morphologically, and syntactically.^30^ Factors such as cultural context, emotionality, untranslatability, and translators need to be considered in cross-language translation.^31^ In this study, we strictly followed the guidance of the World Health Organization’s Process of Translation and Adaptation of Instruments to minimize the impact of the aforementioned factors. Through the translation–back translation process, evaluation by expert panels, and pre-testing, we translated the SaRDS into Chinese with functional equivalence to the original text.

In this study, the translated Chinese version of SaRDS was tested for content validity and face validity in expert panels, and the results showed that the Chinese version of SaRDS was valid and reliable with strong consistency. In the pre-testing, the Chinese version of SaRDS demonstrated good feasibility and took participants about 5 min to complete, suggesting its low response burden. In the psychometric evaluation, this study recruited the CRC and nonclinical general populations. The scores of the CRC population on the 14 subscales and the total score were significantly higher than those of the nonclinical general population, suggesting that the combination treatment of CRC indeed increases the incidence and severity of sexual dysfunction in CRC reproductive-age patients, with a corresponding increase in sexual distress at the intra-personal and relationship levels. In addition, compared with the English version of SaRDS,^14^ the scores of the Chinese nonclinical general population were slightly higher than those of the Australian nonclinical general population. The variations in SaRDS scores between our and previous study may be due to differences in the characteristics of the study samples: the samples in the original SaRDS study were all community-based and skewed toward highly educated and high-functioning individuals.

The CFA showed that the Chinese version of SaRDS contained 14 factors, consistent with the English version of SaRDS. Sexual distress is multi-dimensional and reflects multiple types of distress.^32^ The subscales of the SaRDS cover both intra-personal distress as well as inter-personal distress, including negative cognitions of self (self-esteem, body image, and normalness), negative cognitions of their relationship (security, infidelity, predictability, and relationship quality), inter-personal distress (anxiety, guilt, and hopelessness), and behavioral change in the relationship (conflict, initiation, communication, and physical affection), which provides more extensive information than previous scales with only total scores, such as the SDRDS^13^ and the Female Sexual Distress Scale-Revised.^33^ Since sexual function most often occurs in a dyadic context, relationship factors and processes play a role in sexual outcome,^7^ which suggests that when measuring the distress caused by sexual dysfunction, it is not comprehensive to focus only on the distress at the intra-personal level, and the relationship level also should be considered.

Furthermore, to determine whether the 14-factor structure of the SaRDS is stable and equivalent across different populations and genders, we conducted multi-group confirmatory factor analyses evaluating measurement invariance. Our findings indicated that the SaRDS exhibited satisfactory configural, metric, and scalar invariance across both CRC and nonclinical general populations, as well as across gender groups (male vs. female). These results demonstrate that the factor structure, item loadings, and item intercepts are consistent and meaningful across populations and genders. Thus, the SaRDS can reliably be applied to assess and meaningfully compare sexual and relationship distress across cancer patients, general populations, and across male and female respondents, further supporting its broad utility and robustness as a psychometric tool.

To assess criterion-related validity, the SaRDS was correlated with both the ASEX and QRI, as they are current gold standard measures of sexual distress and relationship satisfaction. The SaRDS had a positive correlation with ASEX and a negative correlation with QRI, which is consistent with the findings in the English version of the SaRDS. As expected, the SaRDS was significantly correlated with existing measures of sexual and relationship distress, indicating that SaRDS is reliable. Finally, the *α* value of the Chinese version of the SaRDS was .865, and the *α* values of the 14 subscales were above .70, indicating good internal consistency.

However, it is important to acknowledge that the correlation between the SaRDS and ASEX in our study was relatively weak (*r* = .230). One plausible explanation for this modest correlation is that the two instruments measure related yet distinct constructs. Specifically, the ASEX primarily evaluates the severity of sexual dysfunction symptoms and associated clinical distress directly linked to these symptoms, whereas the SaRDS assesses broader dimensions of sexual and relationship distress, encompassing emotional and relational aspects that extend beyond specific sexual dysfunction symptoms. Previous research has consistently highlighted that sexual distress involves emotional experiences related to intimacy, relationship dynamics, and subjective perceptions of one's sexuality, which may not always align closely with measures focused solely on symptom severity of sexual dysfunction.^34^ Therefore, the moderate-to-low correlation observed in our study likely reflects conceptual differences between these two instruments rather than a limitation inherent to the SaRDS itself. Future research is needed to further elucidate the nuanced relationship between the severity of sexual symptoms and broader sexual and relationship distress constructs.

### Study limitations

Although our findings provide important support for the psychometric properties of the Chinese version of SaRDS, some methodological limitations may reduce its scope for general applicability. First, we gathered the sample exclusively in the urban areas of Tianjin and Jiangxi, and the clinical samples were only from the CRC population, so whether the results may be generalizable to other locations in China and other clinical samples needs further exploration; a larger and more diverse sample is needed in future research. Second, regarding the validation method, we did not perform test–retest reliability and sensitivity tests. Hence, future investigations should include further evaluation of the psychometric properties of the Chinese version. Third, this study only captured individuals’ responses and couldn’t reflect the sexual and relationship distress of partners. Future research should implement measurements of SaRDS for both members of a couple to determine whether scores can be combined to provide more information. Fourthly, our inclusion criteria restricted participation exclusively to married individuals, thereby excluding unmarried couples from the study. Considering that the original SaRDS was specifically calibrated for couples who had been in a relationship for at least 6 months, and that the QRI was developed primarily for married or cohabiting individuals, this decision to exclude unmarried couples may limit the broader generalizability of our findings. This limitation is particularly noteworthy given the increasing prevalence of stable, committed, and nonmarital relationships in contemporary Chinese society. Future research should therefore aim to include unmarried couples in stable, committed relationships (eg, cohabiting or long-term partnerships) to further enhance the applicability and external validity of the findings. Finally, clinicians should consider the impact of response bias when using SaRDS in applied settings, as individuals may not answer truthfully if they are concerned that their partners may view their answers. Therefore, ensuring that responses to individual items are kept confidential in the clinical context is critical.

### Clinical implications

According to our findings, the Chinese version of the SaRDS is a valid and reliable instrument with robust psychometric properties for assessing individual and relationship distress related to sexual dysfunction in both clinical cancer and nonclinical general populations. Our analyses further demonstrated measurement invariance across different populations (cancer and general populations) and gender groups (male and female). This indicates that the SaRDS measures the same underlying constructs in a consistent manner across diverse groups, allowing clinicians to confidently interpret and compare scores regardless of population type or gender. The 14 domains identified by the SaRDS provide clinicians with detailed and practical insights, enabling more accurate and personalized interventions for individuals and couples experiencing sexual and relationship distress.

## Conclusion

This study translated the SaRDS into Chinese and comprehensively evaluated its psychometric properties and measurement invariance. Our findings demonstrated that the Chinese version of the SaRDS exhibits strong reliability, validity, and responsiveness in non-English-speaking contexts. Furthermore, multi-group confirmatory factor analyses supported the stability and measurement invariance of the SaRDS structure across clinical cancer and nonclinical general populations, as well as across gender groups. These results provide robust evidence that the SaRDS is a reliable and valid instrument for assessing sexual and relationship distress, enabling meaningful comparisons across different populations, genders, and within couples. Therefore, SaRDS can be confidently utilized in real-world clinical practice and research settings, facilitating accurate assessment and tailored interventions for individuals and couples experiencing sexual and relationship distress.

## Supplementary Material

supporting_information_qfaf041

## Preprint

Preprint link: Research Square https://www.researchsquare.com/article/rs-4723813/v1.
