# Supplementary material for: Psychometric evaluation and measurement invariance of the Sexual and Relationship Distress Scale in cancer and nonclinical general reproductive-age populations
Source: Sex Med. 2025 Jun 2;13(3):qfaf041. doi: 10.1093/sexmed/qfaf041 (PMC12128924; doi:10.1093/sexmed/qfaf041)
Supplement: supporting_information_qfaf041 [file supporting_information_qfaf041.docx]

**The 30-item SaRDS (English version and Chinses version)****.**

| **Item** |
| --- |
| 1. I worry about sex even when I am not with my partner  （即使我不在伴侣身边，我也会担心性问题） |
| 2. I feel anxious when I think about our sexual relationship  （当我想到我们的性关系时我会感到焦虑） |
| 3. I am stressed about sex  （我对性有压力） |
| 4. My partner and I get angry with each other  （我和我的伴侣互相生气） |
| 5. My partner and I regularly argue  （我和我的伴侣经常争吵） |
| 6. My partner and I get annoyed with each other over little things  （我和我的伴侣会因为一些小事而互相恼怒） |
| 7. I do not initiate sex with my partner anymore  （我不再主动与我的伴侣发生性行为） |
| 8. I rarely bother to approach my partner for sex  （我很少主动去接近我的伴侣发生性关系） |
| 9. I feel guilty because I cannot sexually satisfy my partner  （我因为无法在性方面满足我的伴侣而感到内疚） |
| 10. I feel guilty for letting my partner down  （我为让我的伴侣失望而感到内疚） |
| 11. I am worried that my partner has been unfaithful  （我担心我的伴侣不忠） |
| 12. I am worried that my partner will be unfaithful  （我担心我的伴侣将会不忠） |
| 13. I feel undesirable to my partner  （我觉得我的伴侣不喜欢我） |
| 14. I feel unattractive to my partner  （我对我的伴侣而言是没有吸引力） |
| 15. Our sex is routine or predictable  （我们的性生活是例行公事） |
| 16. There is not much variety when we have sex  （我们的性生活没有太多变化） |
| 17. My partner and I do not talk about sex  （我和我的伴侣不谈论性） |
| 18. I avoid talking about sex with my partner  （我避免与伴侣谈论性） |
| 19. I am worried that our relationship might end  （我担心我们的关系可能会结束） |
| 20. I am questioning the strength of our relationship  （我对我们关系的牢固感到质疑） |
| 21. We don’t hug and kiss as much as we used to  （我们不像以前那样经常拥抱和亲吻） |
| 22. We are not as physically affectionate as we used to be  （我们的身体不再像以前那样亲密） |
| 23. I wish more effort was made to fix our sexual problems  （我希望付出更多努力来解决我们的性问题） |
| 24. I feel frustrated that I can’t fix our sexual problems  （我因无法解决性问题而感到沮丧） |
| 25. I have lower confidence because of our sexual problems  （由于我们的性问题，我的信心较低） |
| 26. I have lower self-esteem because of our sexual problems  （由于我们的性问题，我的自尊心较低） |
| 27. My relationship has become more like a friendship  （我的关系变得更像是友谊） |
| 28. My partner and I feel more like flat mates or colleagues  （我和我的伴侣感觉更像是室友或同事） |
| 29. I worry there is something wrong with me sexually  （我担心自己在性方面有问题） |
| 30. I do not feel normal when I compare myself sexually to others  （当我与他人进行性方面的比较时，我感觉不正常） |
